# Large-scale association analysis in Asians identifies new susceptibility loci for prostate cancer

**DOI:** 10.1038/ncomms9469

**Published:** 2015-10-07

**Authors:** Meilin Wang, Atsushi Takahashi, Fang Liu, Dingwei Ye, Qiang Ding, Chao Qin, Changjun Yin, Zhengdong Zhang, Koichi Matsuda, Michiaki Kubo, Rong Na, Xiaoling Lin, Haowen Jiang, Shancheng Ren, Jielin Sun, S. Lilly Zheng, Loic Le Marchand, William B. Isaacs, Zengnan Mo, Christopher A. Haiman, Yinghao Sun, Hidewaki Nakagawa, Jianfeng Xu

**Affiliations:** 1 State Key Laboratory of Reproductive Medicine, Nanjing Medical University, Nanjing 210029, China; 2 Department of Molecular and Genetic Toxicology, Key Laboratory of Modern Toxicology of Ministry of Education, School of Public Health, Nanjing Medical University, Nanjing 211166, China; 3 Center for Cancer Genomics, Wake Forest University School of Medicine, Winston-Salem, North Carolina 27157, USA; 4 Laboratory for Statistical Analysis, RIKEN Center for Integrative Medical Sciences, Yokohama 230-0045, Japan; 5 Fudan Institute of Urology, Huashan Hospital, Fudan University, Shanghai 200040, China; 6 Department of Urology, Fudan University Shanghai Cancer Center, Shanghai Medical College, Fudan University, Shanghai 200032, China; 7 Department of Urology, The First Affiliated Hospital of Nanjing Medical University, Nanjing 210029, China; 8 Laboratory of Molecular Medicine, Human Genome Center, Institute of Medical Science, The University of Tokyo, Tokyo 108-8639, Japan; 9 Laboratory for Genotyping Development, RIKEN Center for Integrative Medical Sciences, Yokohama 230-0045, Japan; 10 Department of Urology, Shanghai Changhai Hospital, Second Military Medical University, Shanghai 200433, China; 11 Program for Personalized Cancer Care, NorthShore University HealthSystem, Evanston, Illinois 60201, USA; 12 Department of Epidemiology Program, University of Hawaii Cancer Center, Honolulu, Hawai 96813, USA; 13 Department of Urology, James Buchanan Brady Urologic Institute, Johns Hopkins University School of Medicine, Baltimore, Maryland 21287, USA; 14 Center for Genomic and Personalized Medicine, Guangxi Medical University, Nanning, Guangxi 530021, China; 15 Department of Preventive Medicine, Norris Comprehensive Cancer Center, Keck School of Medicine, University of Southern California, Los Angeles, California 90089, USA; 16 Laboratory for Genome Sequencing Analysis, RIKEN Center for Integrative Medical Sciences, Tokyo 108-8639, Japan

## Abstract

Genome-wide association studies (GWAS) have identified ∼100 genetic loci associated with prostate cancer risk. Less than a dozen of these loci were initially identified from GWAS in two Asian populations, likely because of smaller sample sizes of these individual GWAS in Asians. Here, we conduct a large-scale meta-analysis of two GWAS from the Japanese population (1,583 cases and 3,386 controls) and the Chinese population (1,417 cases and 1,008 controls), followed by replication in three independent sample sets. We identify two independent susceptibility loci for prostate cancer at 11p15.4 (rs12791447, *P*=3.59 × 10^−8^; *PPFIBP2*) and 14q23.2 (rs58262369, *P*=6.05 × 10^−10^; *ESR2*). The mRNA levels of *PPFIBP2* and *ESR2* are differentially expressed in prostate tumours and paired normal tissues. Our study adds two new loci to the limited number of prostate cancer risk-associated variants in Asians and provides important insight into potential biological mechanisms of prostate cancer.

## Abstract (web summary)

Genetic variations influence the risk of prostate cancer. Here, the authors use a meta-analysis of Genome-wide association studies from Asian populations and uncover new susceptibility loci at 11p15.4 and 14q23.2.

## Graphical abstract

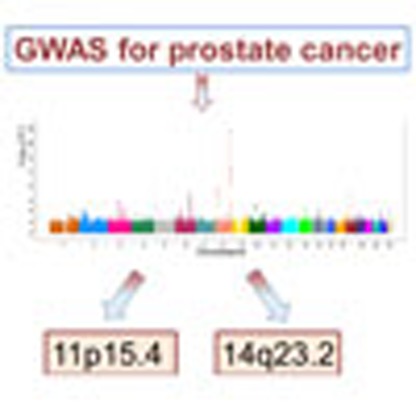

With ∼900,000 new cases diagnosed each year, prostate cancer represents the most frequently diagnosed cancer worldwide in men^1^. The incidence of prostate cancer varies widely between countries and regions, with the highest prevalence rate observed in Western developed countries and the lowest incidence rate observed in Asian countries. In addition to age and race, positive family history is also an established risk factor for prostate cancer, suggesting that inherited genetics may influence the risk for this disease^2^.

To date, genome-wide association studies (GWAS) for prostate cancer have identified a more than 100 susceptibility loci, explaining ∼30% of the familial risk for this disease^3^. However, most of these GWAS were conducted in men of European descent. Only 10 of these loci were initially identified from GWAS in two Asian populations (Japanese and Chinese), likely because of smaller sample sizes of these individual GWAS in Asians^4^,^5^,^6^. It has been demonstrated that combining multiple existing GWAS data using a meta-analysis can increase the statistical power to uncover additional risk loci^7^.

In this study, we performed a large-scale meta-analysis using two existing Asian GWAS data sets to identify novel loci for prostate cancer susceptibility in Asians. In addition, promising single-nucleotide polymorphisms (SNPs) were further evaluated in three independent study populations of Japanese and Chinese descents. We report strong evidence for two prostate cancer susceptibility loci at 11p15.4 and 14q23.2. These results provide additional insight into the inherited genetic basis of prostate cancer and support the presence of considerable genetic heterogeneity between different ethnic groups.

## Results

### Study overview

**Figure 1:**
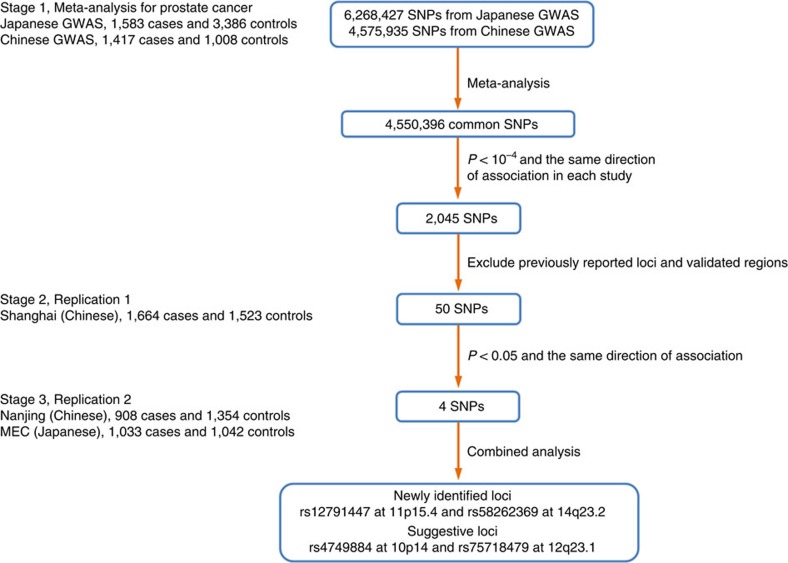
Flow chart of study design and results. The stage 1 included two GWAS from the Japanese prostate cancer GWAS and the Chinese prostate cancer GWAS. The stage 2 consisted of samples from Shanghai. The stage 3 included individuals in two studies from Nanjing and MEC.

**Figure 2:**
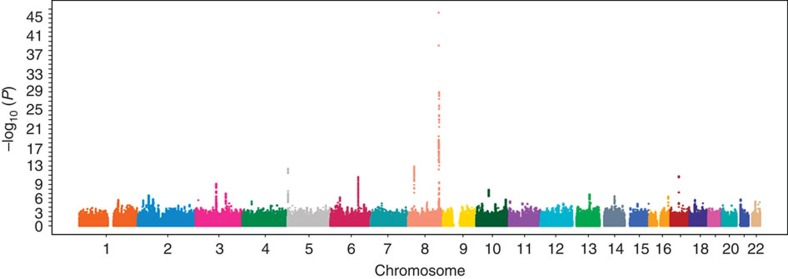
Manhattan plot for meta-analysis results of the prostate cancer GWAS in Japanese and Chinese populations. The *x* axis shows the chromosomal position and the *y* axis shows the −log10 *P*_additive_ value.

The overall study design of the meta-analysis GWAS of prostate cancer is depicted in Fig. 1. After quality-control analysis in the two original GWAS, 1,583 cases and 3,386 controls from the Japanese GWAS and 1,417 cases and 1,008 controls from the Chinese GWAS were retained for further meta-analysis. The characteristics of the subjects are summarized in Supplementary Table 1. After exclusion of rare SNPs that have a minor allele frequency (MAF) less than 5%, a total of 4,550,396 autosomal SNPs were available in both GWAS data sets. A quantile–quantile (Q–Q) plot revealed a modest inflation of the test statistics (*λ*=1.07; Supplementary Fig. 1). The meta-analysis identified multiple previously known genomic regions, with the strongest signal at 8q24 (Fig. 2). Among the 100 known prostate cancer risk-associated SNPs, 33 were significant at *P*_additive_<0.05 (Supplementary Table 2).

### Newly identified susceptibility loci for prostate cancer

To identify novel prostate cancer susceptibility loci, we first filtered out SNPs that had the *P*>1 × 10^−4^ from the meta-analysis or had an opposite direction of association in the two GWAS studies. A total of 2,045 SNPs remained in the analysis (Supplementary Table 3). We then applied the following criteria to select promising novel SNPs: (a) had low linkage disequilibrium (LD; *r*^2^<0.1) with the 100 known prostate cancer risk-associated SNPs; (b) only one SNP with the strongest evidence for association within each LD block was selected; and (c) had not been previously evaluated in follow-up stages of these two individual GWAS. After these steps, 50 SNPs were identified for further replication analysis (Supplementary Table 4).

For replication, we first genotyped these 50 selected SNPs in an independent Chinese population of 1,664 affected subjects and 1,523 control subjects from Shanghai, China (Replication 1). One SNP failed in the assay design and four SNPs failed in the genotyping stage because of poor clusters. Of the remaining 45 SNPs, four SNPs (rs4749884 at 10p14, rs12791447 at 11p15.4, rs75718479 at 12q23.1 and rs58262369 at 14q23.2) showed nominally significant associations at *P*_additive_<0.05 and had the same directions of association as in the GWAS meta-analysis (Supplementary Table 5).

We next examined the four significant SNPs in two independent Asian prostate cancer case–control populations, including 1,941 prostate cancer cases and 2,396 controls (Replication 2a, 908 cases and 1,354 controls of Chinese from Nanjing, China, and Replication 2b, 1,033 cases and 1,042 controls of Japanese descent from Multiethnic Cohort (MEC)). Three of these four SNPs were confirmed in the Nanjing population (*P*_additive_<0.05) and with an effect in the same direction. However, none of the SNPs was confirmed in the MEC population (Table 1).

**Figure 3:**
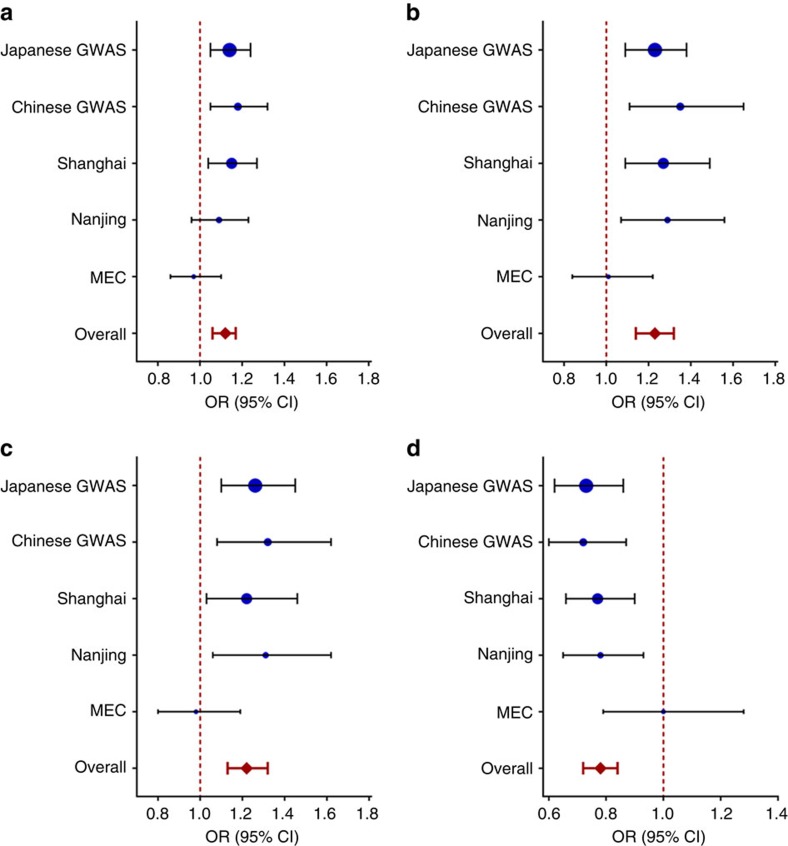
Forest plot of effect size and direction for four SNPs associated with prostate cancer risk. (**a**) rs4749884 at 10p14, (**b**) rs12791447 at 11p15.4, (**c**) rs75718479 at 12q23.1 and (**d**) rs58262369 at 14q23.2. The circle and horizontal lines represent study-specific odds ratio (OR) and 95% confidence interval (CI). The diamond shows the summary OR and 95% CI.

On the basis of our meta-analysis of all independent populations in this study (GWAS stage and confirmation stages), we found that two SNPs were associated with prostate cancer risk at the genome-wide significance level; *P*_additive_=3.59 × 10^−8^ for rs12791447 at 11p15.4 and *P*_additive_=6.05 × 10^−10^ for rs58262369 at 14q23.2. The odds ratios for prostate cancer were 1.23 and 0.78 for the effect allele C of rs12791447 and rs58262369, respectively (Fig. 3). We also identified two loci with suggestive evidence for prostate cancer association, rs4749884 at 10p14 (*P*_additive_=6.68 × 10^−6^) and rs75718479 at 12q23.1 (*P*_additive_=1.12 × 10^−6^). No significant heterogeneity for these four SNPs was observed among different sets of study populations (*P*_heterogeneity_>0.05; Table 1).

We also investigated the associations of these four SNPs with disease aggressiveness among prostate cancer patients. However, we did not find significant associations with aggressiveness of prostate cancer for these four SNPs (*P*_additive_>0.05; Supplementary Table 6).

### Examination of identified susceptibility loci in Europeans

To investigate the association of prostate cancer risk with these identified genetic loci in other ethnicities, we tested the associations in individuals of European descent using data from Cancer Genetic Markers of Susceptibility. No significant association with prostate cancer risk was found for these four SNPs and other SNPs that are in strong LD with these four SNPs (*r*^2^>0.70; Supplementary Table 7).

### Functional annotation and expression analysis of novel loci

**Figure 4:**
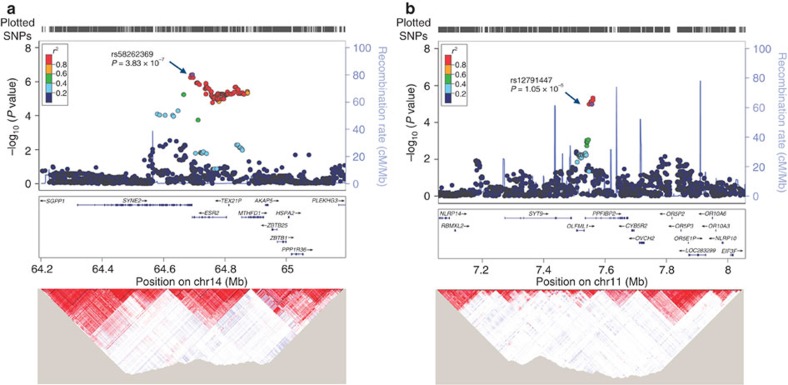
Regional association plot of two loci associated with prostate cancer risk. (**a**) rs58262369 at 14q23.2 and (**b**) rs12791447 at 11p15.4. Top, association results (−log10 *P*) of meta-analysis for each SNP are shown within 500 kb of the index SNP (purple circle). The recombination rate across each region was estimated from the 1000 Genomes Project CHB and JPT populations (version 3, March 2012 release). Bottom, the LD structure (*r*^2^) was derived from Han Chinese in Beijing (CHB) and Japanese in Tokyo (JPT) genotypes in Haploview 4.2, and the colour intensity of each SNP represents the strength of LD. Physical position are based on the NCBI database, build 37.

In the meta-analysis of GWAS, we identified two novel loci associated with prostate cancer, rs58262369 at 14q23.2 (*ESR2*; Fig. 4a) and rs12791447 at 11p15.4 (*PPFIBP2*; Fig. 4b). The mRNA expression levels of genes at these two loci were analysed in prostate tumour tissues and normal tissues (Supplementary Table 8). Examination of rs12791447 using data from Encyclopedia of DNA Elements (ENCODE) revealed significant modification of H3K4me3 (Supplementary Table 9). We also found that rs12791447 was an expression quantitative trait locus (eQTL) for *PPFIBP2*. However, rs58262369 was not an eQTL for *ESR2* or other genes at 14q23.2 (Supplementary Table 10).

## Discussion

In this large-scale association analysis in Asians, we identified two novel prostate cancer susceptibility loci at 11p15.4 and 14q23.2 that reached genome-wide significance level, as well as two additional loci at 10p14 and 12q23.1 with suggestive evidence for association with prostate cancer risk.

It is noted that for two of these four SNPs, rs58262369 at 14q23.2 and rs75718479 at 12q23.1, the genotypes in the two original GWAS were imputed. However, the quality of imputation for these two SNPs was high (see Methods). Furthermore, we genotyped these two SNPs by the TaqMan assay in 1,500 subjects randomly selected from the Chinese GWAS. The concordant rate between imputed and genotyped data was 99.3% for rs58262369 and 99.5% for rs75718479.

The SNP rs58262369 at 14q23.2 is located in the 3′-untranslated region (3′-UTR) of the *ESR2* gene (also known *ESR beta*), which encodes a member of the family of oestrogen receptors (Fig. 4a). Oestrogen plays a vital role in mammary gland development and is also involved in prostate cancer progression, exerting its biological actions through the oestrogen receptors *ESR1* and *ESR2* (refs 8, 9). *ESR2*, originally discovered in a rat prostate cDNA library, has highest expression in normal prostate epithelial cells^10^. *ESR2* knockout mice developed prostatic hyperplasia during the aging process^11^. It has been shown that *ESR2* has antiproliferative and pro-differentiation roles in the prostatic epithelium, reducing prostate cancer risk^9^,^12^. We also found that *ESR2* was upregulated in prostate tumour tissues compared with normal prostate tissues in The Cancer Genome Atlas (TCGA) data (*P*_*t*-test_=0.028; Supplementary Table 8). However, the magnitude of *ESR2* mRNA expression was not high, which should be confirmed via analysis of the expression levels of ESR2 protein in prostate tumour tissues. Several genetic variants in *ESR2*, including rs1256049, have been demonstrated to contribute to prostate cancer risk^13^,^14^. However, this SNP (rs1256049), which is in weak LD with rs58262369 (*r*^2^=0.05), showed no association with prostate cancer in the current study (*P*_additive_=0.149). HaploReg prediction revealed that rs58262369 disrupted the motifs of EWSR1-FLI1 and STAT, indicating that it would have a regulatory impact on *ESR2* (Supplementary Table 9). The SNP rs58262369 also maps to a highly conserved sequence in the 3′-UTR of *ESR2*, which may disturb the regulation of *ESR2* by the binding of microRNA-942 predicted by MirSNP^15^. Our eQTL analysis using TCGA data showed no significant association of rs58262369 with *ESR2* gene expression (*P*_additive_=0.482) in prostate cancer tissues, nor with any other genes within 500 kb of rs58262369 (Supplementary Table 10). It is possible that this variant in the 3′-UTR of *ESR2* is related with efficiency of translation of the ESR2 protein. Further fine-mapping studies are warranted to elucidate the biological role of this association signal at 14q23.2.

The SNP rs12791447 at 11p15.4 is located in an intron of the *PPFIBP2* gene (Fig. 4b). This gene encodes the PTPRF-interacting protein, binding protein 2 (liprin beta 2), which was differentially expressed in endometrial cancer^16^. We also found that the expression level of *PPFIBP2* was significantly lower in tumour tissues than in normal tissues in TCGA data set (*P*_*t*-test_=1.89 × 10^−10^; Supplementary Table 8). Several other genes at this locus, including *CYB5R2*, *EIF3F*, *NLRP10*, *OLFML1* and *OVCH2*, were also found to be significantly expressed in prostate tumours in comparison with normal tissues. In TCGA prostate cancer data, rs12791447 acted as an eQTL for *PPFIBP2* and *NLRP14*, which was consistent with the expression levels of blood-related eQTL results for *PPFIBP2* (Supplementary Table 10). The GM12878 data from ENCODE suggest that rs12791447 maps to a strong enhancer region marked by the histone modification of H3K4me3 (Supplementary Table 9). On the basis of above data, we propose that *PPFIBP2* is the candidate susceptibility gene involved in the association observed at this locus. However, other causal or functional SNPs operating the regulatory mechanisms at this locus cannot be ruled out. It is noted that rs12791447 is ∼530 kb away from two SNPs (rs7126629 and rs7127900) at 11p15.4, which were previously reported to be associated with prostate cancer susceptibility in European and Chinese populations, respectively^17^,^18^. The association of rs12791447 with prostate cancer was not changed after adjusting for either rs7126629 or rs7127900, indicating that it is independent from these two reported SNPs at 11p15.4.

The SNP rs4749884 at 10p14 is in an intergenic region. ENCODE data indicate that alleles at rs4749884 can disrupt the binding of the transcription factor *MAFK*, which suppressed the expression of the *hemeoxygenase-1* (*HO-1*) gene induced by transforming growth factor-β (ref. 19). The SNP rs4749884 is 118 kb away from rs7918885, a SNP that was reported to be associated with prostate cancer in a previous GWAS in West African men^20^. The SNP rs4749884 is not in LD with rs7918885 (*r*^2^=0.002). Meanwhile, rs7918885 and other SNPs in LD with it were not associated with prostate cancer risk in our meta-analysis, suggesting that multiple causal variants may exist at 10p14 in different ethnicities.

The SNP rs75718479 lies at 12q23.1, a region where DNA gain was commonly found in localized prostate tumours of Japanese patients^21^. This region contains a gene of *rhabdomyosarcoma 2-associated transcript* (*RMST*). *RMST* is a newly identified long noncoding RNA, which acts as a transcriptional co-regulator of *SOX2* and is implicated in neurogenesis^22^. Recent studies in mouse models have revealed that *Rmst* was differentially expressed in midbrain dopaminergic neurons in the developing mouse brain^23^. However, the role of *RMST* in prostate cancer is currently unknown. Further studies are warranted to the correlation between rs75718479 and *RMST* in the development of prostate cancer.

It should be noted that four SNPs identified in the meta-analysis GWAS were not replicated in the Japanese descent of the MEC study. The inconsistent results might be because of the differences of lifestyle factors and environment between Japanese individuals in Japan and in the United States, as well as different genetic and environmental risk factors between Japanese and Chinese. Lack of confirmation in the Japanese descent from the MEC population was also reported for SNPs identified in GWAS of Japanese individuals in Japan^4^. Moreover, we did not observe any significant relationship between these four SNPs and prostate cancer risk in the European men. The frequencies of these four SNPs varied among different populations (Supplementary Table 11). For example, rs58262369 at 14q23.2 was not polymorphic in the European population. Further evaluation in other ethnic groups may be needed.

In summary, this study identified two novel susceptibility loci at 11p15.4 and 14q23.2, and two novel loci with suggestive evidence at 10p14 and 12q23.1, which is the largest GWAS to our knowledge on prostate cancer among Asian individuals. Further studies of fine-mapping and functional analyses are warranted to identify and characterize the causative variants for prostate tumorigenesis.

## Methods

### Study populations

The meta-analysis of GWAS in this study was based on two GWAS on prostate cancer in Japan and China^5^,^6^. The Japanese prostate cancer GWAS included 1,583 cases and 3,386 controls from the BioBank Japan, which was established in the Institute of Medical Science at the University of Tokyo^24^. From the BioBank Japan, prostate cancer cases were selected according to the pathological evaluation of biopsy. Among the selected subjects, 229 individuals had a positive family history, and 952 cases had high Gleason grade score. Controls in the Japanese GWAS were also selected from BioBank Japan for other diseases as well as healthy subjects from the Osaka-Midosuji Rotary Club (Osaka, Japan). The Chinese prostate cancer GWAS comprised 1,417 prostate cancer cases and 1,008 controls who are part of the ChinaPCa (ref. 25). All recruited subjects self-reported being Han Chinese. Cases were collected from local hospitals and were newly diagnosed with pathologically confirmed prostate cancer. Control subjects were healthy individuals recruited from community-based studies or while receiving a physical examination in the hospital. Control subjects were excluded if they had an abnormal prostate-specific antigen level higher than 4.0 ng ml^−1^ or positive digital rectal examination.

Three additional study populations (Replications 1, 2a and 2b) were used to validate the suggestive associations from the meta-analysis of GWAS. Individuals in Replications 1 and 2a were Chinese and consisted of 1,664 cases and 1,523 controls from Shanghai and 908 cases and 1,354 controls from Nanjing. Most subjects have been used in previously published association studies^25^,^26^,^27^. Cases were histopathologically confirmed as having prostate cancer, and the controls were all cancer-free males from Shanghai, Nanjing and surrounding areas. Replication 2b was men of Japanese descent from the MEC, a large population-based prospective study including more than 215,000 adults in Hawaii and California^28^. The GWAS of prostate cancer in the MEC study included 1,033 cases and 1,042 controls. The Cancer Genetic Markers of Susceptibility prostate cancer GWAS was a nested case–control study, including 1,172 cases and 1,157 controls, which was part of the Prostate, Lung, Colon and Ovarian Cancer Screening Trial^29^. All participants provided informed consent. This study was approved by the institutional review boards of the Fudan University, Nanjing Medical University and the University of Tokyo and RIKEN Yokohama Institute.

### Genotyping and quality control

The Japanese GWAS was conducted using the Illumina Human610-Quad BeadChip for cases and HumanHap550v3 BeadChip for controls, and the Chinese GWAS was genotyped using the Illumina Human OmniExpress BeadArray. In addition to quality-control procedures performed in previous studies, SNPs with genotyping rate of<95%, MAF<0.05 or *P*<0.001 in a Hardy–Weinberg Equilibrium test were further removed before imputation analysis. After exclusion of imputed SNPs with MAF<0.05 or information score (INFO)<0.5, 6,268,427 and 4,575,935 SNPs remained for Japanese GWAS and Chinese GWAS, respectively. A total of 4,550,396 SNPs were available in both studies and used for further meta-analysis. We selected all SNPs with *P*<1.0 × 10^−4^ for the Replication 1 study. Genotyping for Replication 1 was conducted using the Sequenom MassARRAY platform, and genotyping for Replication 2a was conducted using the TaqMan assays. The primer and probe sequences are shown in Supplementary Table 12. On each 96-well plate, two negative controls (water) and two blinded duplicates were included as quality-control samples. Genotyping was performed independently by two researchers in a blinded manner. For *in silico* replication of the MEC study, genotyping of the subjects was conducted using the Illumina Human660W bead array^30^.

### Functional annotation

Genomic annotations on the index SNPs and surrogates were identified using the ENCODE data. The threshold of LD was *r*^2^>0.80 on the basis of Asians from the 1000 Genomes Project. HaploReg 2.0 (http://www.broadinstitute.org/mammals/haploreg/haploreg.php/) was used to query the histone marks, DNase I hypersensitivity, the binding of transcription factor and motif instances^31^. RegulomeDB (http://regulome.stanford.edu/) was then used to explore potential functional annotations for the genomic regions surrounding the index SNPs^32^. The high score of RegulomeDB indicated a relatively high degree of evidence for potential regulatory function. Furthermore, SNPs in 3′-UTR of gene were predicted using MirSNP (http://cmbi.bjmu.edu.cn/mirsnp) to test the changes in microRNA-binding sites^15^.

### Expression analysis

We downloaded the mRNA expression profiles from TCGA project (http://cancergenome.nih.gov/) by RNA-Seq (level 3). A total of 497 prostate tumour tissues and 115 normal prostate tissues were available on 10 January 2015. The reads per kilobase per million was used to quantify of gene expression levels. The expression levels of mRNA were normalized by using log2 transformation, and genes with zero value or missing data in any sample were removed. Differential expression of genes for each gene was measured between tumour and normal tissues.

For each individual in the TCGA data, we also obtained level 2 SNP data from prostate tumour tissues, normal tissues and blood, which were genotyped using Affymetrix SNP Array 6.0 chips. SNPs within 500 kb of index SNP were extracted from genotyped or imputed genotypes. The eQTL analysis was measured only in tumour tissues with mRNA expression. We also queried the correlation between variants and candidate gene expression from the Blood eQTL Brower^33^.

### Statistical analysis

SNPs that passed quality control were imputed on the basis of data from the 1000 Genomes Project (version 3, March 2012 release) using IMPUTE2 (ref. 34). Only imputed genotypes of high quality (INFO>0.50) were included in further association analysis. The associations in Japanese GWAS and Chinese GWAS were first analysed under an additive model using PLINK1.07. The odds ratio, 95% CI, the corresponding s.e. and *P* value for each SNP were obtained from the logistic regression model. The meta-analysis of two GWAS data was performed using a fixed-effect model and weighted by the estimated SE using GWAMA (ref. 35). The heterogeneity of studies was tested by the Cochran's Q statistic and *I*^2^. If the test of heterogeneity existed (*P*<0.01), the random-effect model was provided. The LD structure (*r*^2^) was calculated using Haploview 4.2 (ref. 36). Expressed differences in each gene between tumours and normal samples were measured by the Student's *t*-test. The relationships of SNP genotypes with gene expression levels were evaluated using a linear regression model.

## Additional information

**How to cite this article:** Wang, M. *et al*. Large-scale association analysis in Asians identifies new susceptibility loci for prostate cancer. *Nat. Commun.* 6:8469 doi: 10.1038/ncomms9469 (2015).

## Supplementary Material

Supplementary Information Supplementary Figure 1 and Supplementary Tables 1-12

## Figures and Tables

**Table 1:** Summary results of meta-analysis and replication studies for prostate cancer.

**SNP**	Allele*	**Chr**	**Position**†	**Gene**‡	**Study**	**Population**	**EAF**§	**OR**	***P* value**	***P***_**het**_||
rs4749884	C/A	10p14	9644800	—	Japanese GWAS	Japanese	0.533	1.14	2.29E−03	
					Chinese GWAS	Chinese	0.434	1.18	6.49E−03	
					Meta-analysis GWAS			1.15	4.74E−05	0.673
					Shanghai	Chinese	0.437	1.15	4.87E−03	
					Nanjing	Chinese	0.446	1.09	1.68E−01	
					MEC	Japanese	0.460	0.97	6.41E−01	
					Meta-analysis of all studies			1.12	6.68E−06	0.100
rs12791447	C/T	11p15.4	7556577	*PPFIBP2*	Japanese GWAS	Japanese	0.136	1.23	7.66E−04	
					Chinese GWAS	Chinese	0.086	1.35	3.13E−03	
					Meta-analysis GWAS			1.26	1.05E−05	0.421
					Shanghai	Chinese	0.098	1.27	2.73E−03	
					Nanjing	Chinese	0.095	1.29	8.89E−03	
					MEC	Japanese	0.138	1.01	8.97E−01	
					Meta-analysis of all studies			1.23	3.59E−08	0.177
rs75718479	C/A	12q23.1	97876906	*RMST*	Japanese GWAS	Japanese	0.877	1.26	6.50E−04	
					Chinese GWAS	Chinese	0.910	1.32	7.75E−03	
					Meta-analysis GWAS			1.28	1.71E−05	0.732
					Shanghai	Chinese	0.906	1.22	2.49E−02	
					Nanjing	Chinese	0.902	1.31	1.35E−02	
					MEC	Japanese	0.887	0.98	8.23E−01	
					Meta-analysis of all studies			1.22	1.12E−06	0.111
rs58262369	C/T	14q23.2	64693912	*ESR2*	Japanese GWAS	Japanese	0.928	0.73	1.40E−04	
					Chinese GWAS	Chinese	0.889	0.72	8.04E−04	
					Meta-analysis GWAS			0.73	3.83E−07	0.929
					Shanghai	Chinese	0.898	0.77	1.05E−03	
					Nanjing	Chinese	0.898	0.78	7.38E−03	
					MEC	Japanese	0.929	1.00	9.87E−01	
					Meta-analysis of all studies			0.78	6.05E−10	0.149

EAF, effect allele frequency; GWAS, genome-wide association study; MEC, Multiethnic Cohort; OR, odds ratio; SNP, single-nucleotide polymorphism.

^*^Effect/non-effect allele.

^†^On the basis of the National Center for Biotechnology Information (NCBI) database, build 37.

^‡^Nearby gene.

^§^Effect allele frequency in the controls.

^||^*P* value of Cochran's *Q*-test for the heterogeneity.
