# Supplementary material for: Large-scale association analysis in Asians identifies new susceptibility loci for prostate cancer
Source: Nat Commun. 2015 Oct 7;6:8469. doi: 10.1038/ncomms9469 (PMC4633711; doi:10.1038/ncomms9469)
Supplement: Supplementary Information — Supplementary Figure 1 and Supplementary Tables 1-12 [file ncomms9469-s1.pdf]

Supplementary Figure 1. Quantile-quantile (Q-Q) plot of the meta-analysis restuls. The genomic inflation factor (λ) in the meta-analysis GWAS was 1.07.

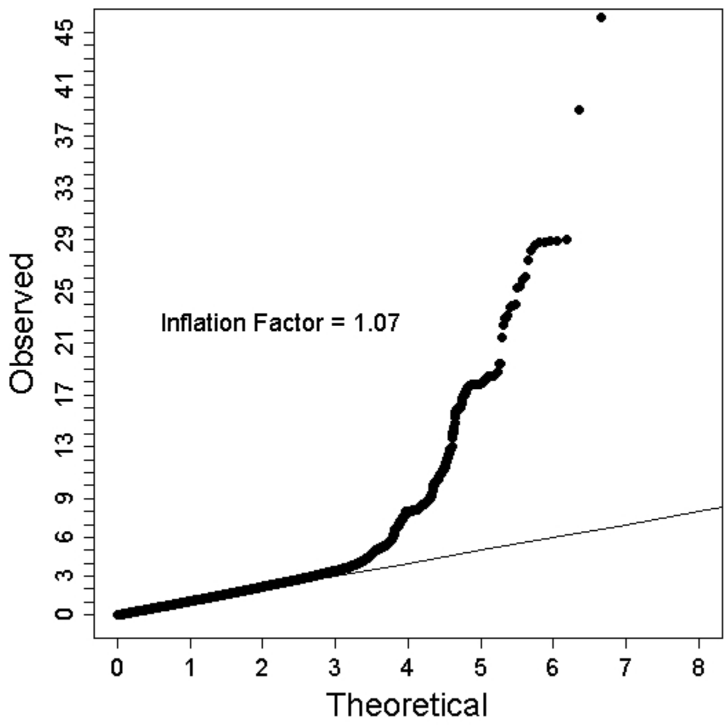

Supplementary Table 1. Description of study participants and genotyping method

Design	Study	Population	Genotyping	Sample size		Age (mean ± SD)		Gleason score (%)[a]		Aggressiveness (%)[a]	
				**Cases**	**Controls**	**Cases**	**Controls**	**<7**	**≥7**	**Aggressive**	**Non-aggressive**
Stage 1, Discovery	Japanese GWAS	Japanese	Illumina Human610 (case)/HumanHap550v3 (control)	1,583	3,386	69.3 ± 7.4	52.5 ± 15.1	355 (27.2)	952 (72.8)	NA	NA
	Chinese GWAS	Chinese	Illumina Human OmniExpress	1,417	1,008	71.3 ± 8.1	62.1 ± 10.0	355 (26.3)	993 (73.7)	1,010 (74.7)	342 (25.3)
Stage 2, Replication 1	Shanghai	Chinese	MassARRAY iPLEX Sequenom	1,664	1,523	70.1 ± 7.7	67.9 ± 6.3	631 (55.3)	510 (44.7)	610 (53.1)	538 (46.9)
Stage 3, Replication 2	Nanjing	Chinese	TaqMan	908	1,354	71.5 ± 6.9	67.4 ± 7.6	301 (33.8)	589 (66.2)	611 (68.3)	284 (31.7)
	MEC	Japanese	Illumina Human660W	1,033	1,042	64.0 ± 7.5	63.9 ± 7.5	NA	NA	378 (47.6)	416 (52.4)
Total				6,605	8,313						

[a]Some cases were not available for the clinical information.

NA, not available

Supplementary Table 2. Meta-analysis of reported prostate cancer susceptibility loci with two prostate cancer GWASs

Chr	SNP	Locus	Position[a]	Gene[b]	Reported SNPs			Meta-analysis				
					RA[c]	OR	*P*	Allele[d]	OR	*P*	P_{het}[e]	I^2
1	rs17599629	1q21	150658287	*GOLPH3L*	G	1.10	5.90E-11	A/G	1.01	8.85E-01	0.314	0.013
1	rs1218582	1q23.1	154834183	*KCNN3*	G	1.06	1.95E-08	NA				
1	rs4245739	1q32.1	204518842	*MDM4*	A	1.10	2.01E-11	A/C	1.03	7.49E-01	0.030	0.788
1	rs1775148	1q32.1	205757824	*SLC41A1*	C	1.12	2.00E-03	NA				
2	rs11902236	2p25.1	10117868	*GRHL1*	T	1.07	2.84E-08	C/T	1.04	4.80E-01	0.463	0.000
2	rs9287719	2p25	10710730	*NOL10*	C	1.07	1.80E-08	T/C	0.93	5.25E-02	0.621	0.000
2	rs13385191	2p24.1	20888265	*C2orf43*	G	1.15	7.50E-08	G/A	1.17	4.73E-06	0.151	0.516
2	rs1465618	2p21	43553949	*THADA*	T	1.27	1.60E-08	NA				
2	rs721048	2p15	63131731	*EHBP1*	A	1.15	7.66E-09	G/A	0.90	2.46E-01	0.165	0.481
2	rs10187424	2p11.2	85794297	*VAMP8*	T	1.19	3.10E-15	C/T	0.89	1.78E-03	0.206	0.375
2	rs12621278	2q31.1	173311553	*ITGA6*	A	1.35	8.70E-23	C/T	0.89	5.37E-03	0.744	0.000
2	rs2292884	2q37.3	238443226	*MLPH*	G	1.14	4.30E-08	G/A	1.09	2.37E-02	0.056	0.726
2	rs3771570	2q37.3	242382864	*FARP2*	T	1.12	5.22E-09	C/T	1.05	4.09E-01	0.772	0.000
3	rs2660753	3p12.1	87110674	*VGLL3*	C	1.18	2.70E-08	C/T	0.84	5.51E-06	0.253	0.236
3	rs2055109	3p11.2	87467332	*POU1F1*	C	1.20	3.94E-08	C/T	1.29	1.05E-05	0.408	0.000
3	rs7611694	3q13.2	113275624	*SIDT1*	A	1.10	3.80E-13	G/T	0.91	2.96E-02	0.654	0.000
3	rs10934853	3q21.3	128038373	*EEFSEC*	A	1.12	2.90E-10	G/T	0.94	9.17E-02	0.971	0.000
3	rs6763931	3q23	141102833	*ZBTB38*	A	1.18	2.00E-08	G/A	0.9	2.28E-03	0.557	0.000
3	rs10936632	3q26	170130102	*CLDN11/SKIL*	A	1.14	6.60E-12	NA				
4	rs10009409	4q13	73855253	*COX18*	T	1.09	2.10E-10	T/C	1.04	3.32E-01	0.026	0.798
4	rs1894292	4q13.3	74349158	*AFM*	G	1.10	5.02E-13	NA				
4	rs17021918	4q22.3	95562877	*PDLIM5*	C	1.19	4.20E-15	G/A	1.04	2.53E-01	0.882	0.000
4	rs7679673	4q24	106061534	*TET2*	C	1.19	2.60E-14	A/C	0.86	6.10E-04	0.972	0.000
5	rs12653946	5p15.33	1895829	*IRX4*	T	1.26	3.90E-18	G/A	0.78	1.94E-12	0.499	0.000
5	rs2121875	5p12	44365545	*FGF10*	C	1.09	4.00E-08	G/T	0.98	5.49E-01	0.726	0.000
5	rs6869841	5q35.2	172939426	*LOC285593*	T	1.07	4.63E-08	C/T	0.98	7.21E-01	0.765	0.000
6	rs4713266	6p24	11219030	*NEDD9*	C	1.07	3.90E-08	T/C	0.94	1.51E-01	0.629	0.000

6	rs115457135	6p22	30073776	*TRIM31*	A	1.08	1.90E-08	NA				
6	rs130067	6p21	31118511	*CCHCR1*	G	1.20	3.20E-08	C/A	1.02	6.80E-01	0.543	0.000
6	rs3096702	6p21.3	32192331	*NOTCH4*	A	1.07	4.78E-09	NA				
6	rs115306967	6p12	32400939	*HLA-DRB6*	G	1.08	2.70E-09	NA				
6	rs1983891	6p21.1	41536427	*FOXP4*	T	1.15	7.60E-08	G/A	0.86	5.15E-05	0.013	0.837
6	rs9443189	6q14	76495882	*MYO6*	G	1.07	8.00E-02	A/G	1.06	1.05E-01	0.233	0.298
6	rs2273669	6q21	109285189	*ARMC2/SESN1*	G	1.07	7.91E-09	C/T	0.89	7.43E-02	0.629	0.000
6	rs339331	6q22.2	117210052	*RFX6*	T	1.22	1.60E-12	C/T	0.78	4.27E-11	0.885	0.000
6	rs1933488	6q25.2	153441079	*RGS17*	A	1.12	4.34E-18	C/T	0.97	5.66E-01	0.831	0.000
6	rs9364554	6q25.3	160833664	*SLC22A3*	T	1.17	5.50E-10	C/T	1.02	6.65E-01	0.073	0.689
7	rs12155172	7p15.3	20994491	*RPL23P8*	A	1.11	4.95E-13	G/A	0.93	7.93E-02	0.699	0.000
7	rs10486567	7p15.2	27976563	*JAZF1*	G	1.19	2.14E-06	C/T	1.14	1.63E-02	0.282	0.136
7	rs56232506	7p12	47437244	*TNS3*	A	1.07	1.80E-09	NA				
7	rs6465657	7q21.3	97816327	*LMTK2*	C	1.12	1.10E-09	C/T	1.07	2.07E-01	0.319	0.000
8	rs2928679	8p21.2	23438975	*SLC25A37*	A	1.53	7.10E-08	G/A	0.9	5.70E-02	0.773	0.000
8	rs1512268	8p21.2	23526463	*NKX3-1*	T	1.23	3.40E-30	C/T	0.76	1.40E-13	0.429	0.000
8	rs11135910	8p21.2	25892142	*EBF2*	T	1.11	8.16E-11	G/A	1.08	4.48E-01	0.693	0.000
8	rs10086908	8q24.21	128011937	*POU5F1B*	T	1.25	7.90E-08	G/A	0.83	2.24E-05	0.076	0.683
8	rs16901979	8q24.21	128124916	*POU5F1B*	A	1.79	1.10E-12	C/A	0.72	1.00E-16	0.895	0.000
8	rs16902094	8q24.21	128320346	*POU5F1B*	G	1.21	6.20E-15	NA				
8	rs620861	8q24.21	128335673	*POU5F1B*	G	1.28	4.80E-08	G/A	1.13	5.55E-04	0.315	0.008
8	rs6983267	8q24.21	128413305	*POU5F1B*	G	1.58	9.42E-13	G/T	1.23	8.36E-09	0.891	0.000
8	rs1447295	8q24.21	128485038	*LOC727677*	A	2.23	1.53E-14	C/A	0.65	3.92E-23	0.141	0.539
9	rs17694493	9p21	22041998	*CDKN2B-AS1*	G	1.10	4.00E-08	NA				
9	rs817826	9q31.2	110156300	*KLF4*	T	1.41	5.45E-14	G/A	1.22	4.16E-03	0.045	0.751
9	rs1571801	9q33.2	124427373	*DAB2IP*	T	1.36	2.84E-05	C/A	0.98	8.35E-01	0.645	0.000
10	rs76934034	10q11	46082985	*MARCH8*	T	1.14	4.80E-09	NA				
10	rs10993994	10q11	51549496	*MSMB*	T	1.57	7.31E-13	C/T	0.83	3.58E-08	0.074	0.686
10	rs3850699	10q24.32	104414221	*TRIM8*	A	1.10	4.87E-10	C/T	0.91	9.93E-02	0.586	0.000
10	rs2252004	10q26.12	122844709	*WDR11*	C	1.16	1.98E-08	NA				

10	rs4962416	10q26	126696872	*CTBP2*	C	1.46	1.70E-07	NA				
11	rs7127900	11p15.5	2233574	*TH*	A	1.28	2.70E-33	G/A	1.02	7.06E-01	0.082	0.669
11	rs1938781	11q12.1	58915110	*FAM111A*	G	1.16	1.10E-10	G/A	1.17	1.69E-05	0.256	0.224
11	rs12418451	11q13.2	68935419	*TPCN2*	A	1.36	1.20E-06	G/A	0.91	2.48E-01	0.859	0.000
11	rs10896449	11q13	68994667	*MYEOV*	G	1.41	1.76E-09	C/T	1.2	1.27E-02	0.914	0.000
11	rs11568818	11q22.3	102401661	*MMP7*	T	1.10	1.56E-11	G/A	0.96	4.97E-01	0.648	0.000
11	rs11214775	11q23	113807181	*HTR3*	G	1.08	3.00E-08	G/A	1.05	2.45E-01	0.794	0.000
12	rs80130819	12q13	48419618	*RP1/SENP1*	A	1.13	4.30E-08	NA				
12	rs10875943	12q13.13	49676010	*PRPH*	C	1.18	6.90E-12	C/T	1.1	4.68E-02	0.386	0.000
12	rs902774	12q13.2	53273904	*KRT8*	A	1.17	4.70E-09	NA				
12	rs1270884	12q24.21	114685571	*TBX5*	A	1.07	6.75E-11	NA				
13	rs9600079	13q22.1	73728139	*KLF5*	T	1.18	2.80E-09	G/T	0.84	1.01E-06	0.998	0.000
14	rs8008270	14q22.2	53372330	*FERMT2*	C	1.12	1.78E-14	NA				
14	rs7153648	14q23	61122526	*SIX1*	C	1.17	1.40E-04	G/C	0.87	8.81E-04	0.676	0.000
14	rs7141529	14q24.1	69126744	*ZFP36L1*	C	1.09	2.77E-10	C/T	1.07	1.74E-01	0.925	0.000
14	rs8014671	14q24	71092256	*TTC9*	G	1.07	1.30E-08	G/A	1.05	2.02E-01	0.314	0.012
16	rs12051443	16q22	71691329	*PHLPP2*	A	1.10	2.00E-02	A/G	1.04	3.42E-01	0.964	0.000
17	rs684232	17p13.3	618965	*FAM57A*	C	1.10	5.17E-15	C/T	1.08	3.34E-02	0.441	0.000
17	rs11649743	17q21.2	36074979	*HNF1B*	G	1.50	1.70E-09	G/A	1.18	1.86E-05	0.057	0.725
17	rs4430796	17q21.2	36098040	*HNF1B*	A	1.22	1.40E-11	G/A	0.81	5.56E-08	0.032	0.782
17	rs11650494	17q21.33	47345186	*FLJ40194*	A	1.15	1.97E-09	NA				
17	rs7210100	17q21.32	47436749	*ZNF652*	A	1.51	3.40E-13	NA				
17	rs1859962	17q25.1	69108753	*KCNJ2*	G	1.20	2.50E-10	G/T	1.04	2.84E-01	0.095	0.641
18	rs7241993	18q23	76773973	*SALL3*	C	1.09	2.19E-09	C/T	1.07	6.93E-02	0.498	0.000
19	rs8102476	19q13.11	38735613	*PPP1R14A*	C	1.12	1.60E-11	C/T	1.03	4.33E-01	0.562	0.000
19	rs887391	19q13.12	41985624	*C19orf69*	T	1.15	3.20E-07	C/T	0.95	1.60E-01	0.689	0.000
19	rs2735839	19q13.32	51364623	*KLK3*	G	1.20	1.50E-18	C/T	1.08	2.96E-02	0.045	0.751
19	rs103294	19q13.33	54797848	*LILRA3*	C	1.28	5.34E-16	C/T	1.1	2.06E-02	0.005	0.872
20	rs12480328	20q13	49527922	*ADNP*	T	1.30	7.70E-04	T/C	1.16	3.74E-02	0.608	0.000
20	rs2427345	20q13.33	61015611	*C20orf151*	C	1.06	3.64E-08	C/T	0.96	3.11E-01	0.659	0.000

20	rs6062509	20q13.33	62362563	*ZGPAT*	T	1.12	3.57E-16	G/T	0.93	6.63E-02	0.928	0.000
21	rs1041449	21q22	42901421	*TMPRSS2*	G	1.02	7.90E-01	NA				
22	rs2238776	22q11	19757892	*TBX1*	G	1.08	3.00E-02	NA				
22	rs9623117	22q13.2	40452119	*TNRC6B*	C	1.18	4.96E-07	C/T	1.01	9.09E-01	0.425	0.000
22	rs5759167	22q13.31	43500212	*BIK*	G	1.14	5.90E-29	C/A	1.15	1.87E-04	0.515	0.000
X	rs2405942	Xp22.2	9814135	*SHROOM2*	A	1.14	2.37E-10	NA				
X	rs5945619	Xp11.22	51241672	*NUDT11*	C	1.19	1.50E-09	NA				
X	rs5919432	Xq12	67021550	*AR*	A	1.09	1.20E-08	NA				
X	rs2807031	Xp11	52896949	*XAGE3*	C	1.07	8.50E-10	NA				
X	rs6625711	Xq13	70139850	*SLC7A*	A	1.07	6.30E-12	NA				
X	rs4844289	Xq13	70407983	*NLGN3/BCYRN*	G	1.05	1.30E-09	NA				

[a]Based on the NCBI database, build 37.

[b]Neaby gene.

[c]Previously reported risk allele.

[d]Effect/non-effect allele.

[e]*P* value of Cochran's Q-test for the heterogeneity.

NA, not available in the meta-analysis.

Supplementary Table 3. Distribution of the $P_{additive}$ values (< 1.0 x 10^{-4}) and r^2 from the meta-analysis results

LD[a]	P < 1 x 10^{-7}	1 x 10^{-7} ≤ P < 1 x 10^{-6}	1 x 10^{-6} ≤ P < 1 x 10^{-5}	1 x 10^{-5} ≤ P < 1 x 10^{-4}	Sum
$0<r^2\leq0.1$	13	18	268	675	974
$0.1<r^2\leq0.2$	6	10	3	20	39
$0.2<r^2\leq0.3$	2	3	2	7	14
$r^2>0.3$	546	87	207	178	1,018
Sum	567	118	480	880	2,045

[a]Linkage disequilibrium between the top SNPs with P < 10^{-4} with any of the reported prostate cancer loci.

Supplementary Table 4. Summary of top 50 SNPs from meta-analysis of prostate cancer in Chinese and Japanese ($P_{additive} < 1.0 \times 10^{-4}$)

Chr	SNP	Position[a]	Meta-analysis					Results in Chinese						Results in Japanese					
			Allele[b]	OR	P[c]	P_{het}[d]	I^2	Allele[b]	EAF[e]	OR	P	Type	Info[f]	Allele[b]	EAF[e]	OR	P	Type	Info[f]
1	rs76014269	180923595	G/A	1.28	3.84E-05	0.796	0.000	G/A	0.876	1.26	1.19E-02	Imputed	0.96	G/A	0.904	1.30	1.01E-03	Imputed	0.97
1	rs11120266	214359179	T/C	1.16	3.08E-05	0.317	0.000	C/T	0.466	0.82	8.08E-04	Genotyped	-	T/C	0.631	1.13	7.15E-03	Imputed	0.96
2	rs6753841	34272597	A/C	0.84	3.14E-05	0.636	0.000	A/C	0.843	0.81	1.05E-02	Imputed	0.98	A/C	0.754	0.85	8.08E-04	Imputed	0.99
2	rs13031082	51280251	A/G	1.21	6.36E-05	0.688	0.000	A/G	0.838	1.24	7.08E-03	Imputed	1.01	A/G	0.825	1.19	3.03E-03	Imputed	1.00
2	rs354211	54932516	C/T	1.17	3.41E-05	0.429	0.000	A/G	0.217	0.81	4.23E-03	Genotyped	-	C/T	0.652	1.15	1.92E-03	Genotyped	-
2	rs34824338	169934114	C/T	1.22	3.94E-05	0.946	0.000	C/T	0.852	1.22	1.49E-02	Imputed	0.97	C/T	0.827	1.22	8.65E-04	Imputed	0.99
2	rs59817105	196486923	T/C	0.87	6.42E-05	0.470	0.000	C/T	0.367	1.20	3.50E-03	Imputed	1.00	T/C	0.513	0.88	4.78E-03	Imputed	0.95
2	rs11886390	216924139	T/A	1.18	2.90E-05	0.095	0.640	T/A	0.777	1.30	2.02E-04	Imputed	1.01	T/A	0.692	1.13	1.09E-02	Imputed	1.00
3	rs138301420	151135814	A/G	0.60	4.95E-05	0.107	0.615	A/G	0.981	0.81	3.42E-01	Imputed	0.98	A/G	0.981	0.52	1.95E-05	Imputed	0.94
4	rs74832629	38385467	G/A	1.18	4.84E-06	0.726	0.000	G/A	0.588	1.20	3.14E-03	Imputed	0.95	G/A	0.569	1.17	5.20E-04	Imputed	0.96
4	rs35613641	119639182	C/T	0.84	4.04E-05	0.358	0.000	C/T	0.776	0.88	8.23E-02	Imputed	0.98	C/T	0.808	0.81	1.41E-04	Imputed	0.98
4	rs4446264	145137401	A/C	1.18	3.53E-05	0.128	0.568	A/C	0.720	1.28	2.27E-04	Imputed	0.98	A/C	0.721	1.13	1.55E-02	Imputed	0.97
5	rs13154478	161749087	A/G	1.37	3.01E-05	0.883	0.000	G/A	0.091	0.74	5.04E-03	Genotyped	-	A/G	0.931	1.38	2.03E-03	Imputed	0.72
6	rs12192568	27740438	C/A	0.65	3.09E-05	0.431	0.000	A/C	0.012	1.83	1.54E-02	Genotyped	-	C/A	0.958	0.68	4.74E-04	Imputed	0.84
6	rs9351810	72103444	C/T	0.87	8.62E-05	0.890	0.000	C/T	0.581	0.87	2.69E-02	Imputed	0.95	C/T	0.622	0.86	1.19E-03	Imputed	0.98
6	rs73773204	123952569	T/C	1.21	8.85E-05	0.090	0.653	T/C	0.825	1.10	2.27E-01	Imputed	1.00	T/C	0.855	1.30	3.91E-05	Imputed	0.98
7	rs10274959	33218314	T/C	1.36	6.70E-05	0.393	0.000	T/C	0.913	1.28	1.65E-02	Imputed	1.01	T/C	0.956	1.46	9.35E-04	Imputed	0.99
7	rs55932730	147551667	G/C	0.87	7.73E-05	0.377	0.000	G/C	0.547	0.91	9.54E-02	Imputed	1.02	G/C	0.476	0.85	2.01E-04	Imputed	0.99
8	rs2167065	76560454	G/A	1.16	2.73E-05	0.770	0.000	C/T	0.659	1.18	8.94E-03	Imputed	0.94	G/A	0.569	1.16	9.83E-04	Genotyped	-
8	rs10282777	78068366	G/A	0.84	1.12E-05	0.606	0.000	A/G	0.215	1.23	3.40E-03	Genotyped	-	G/A	0.754	0.85	9.05E-04	Genotyped	-
9	rs13300882	101561046	G/A	0.83	3.60E-05	0.144	0.532	G/A	0.875	0.74	9.35E-04	Imputed	0.99	G/A	0.776	0.86	3.86E-03	Imputed	0.95
10	rs11251231	2454195	C/T	0.87	8.12E-05	1.000	0.000	A/G	0.377	1.15	1.97E-02	Genotyped	-	C/T	0.613	0.87	1.50E-03	Genotyped	-
10	rs4749884	9644800	C/A	1.15	4.74E-05	0.673	0.000	C/A	0.434	1.18	6.49E-03	Genotyped	-	C/A	0.533	1.14	2.29E-03	Genotyped	-
10	rs2152433	25152336	C/T	0.85	4.03E-05	0.762	0.000	A/G	0.235	1.19	1.14E-02	Genotyped	-	C/T	0.683	0.86	1.14E-03	Imputed	0.97
10	rs77598054	80244583	G/A	1.23	2.08E-05	0.259	0.214	G/A	0.894	1.35	1.64E-03	Imputed	0.98	G/A	0.822	1.19	2.21E-03	Imputed	0.99
11	rs12791447	7556577	C/T	1.26	1.05E-05	0.421	0.000	G/A	0.086	1.35	3.13E-03	Genotyped	-	C/T	0.136	1.23	7.66E-04	Genotyped	-
11	rs2015997	70664218	C/A	1.25	6.46E-05	0.232	0.299	C/A	0.090	1.38	9.65E-04	Genotyped	-	C/A	0.103	1.20	1.04E-02	Genotyped	-

11	rs11603610	80668994	T/G	0.64	1.59E-05	0.806	0.000	T/G	0.955	0.62	3.38E-03	Imputed	0.85	T/G	0.958	0.66	1.46E-03	Imputed	0.60
11	rs678618	95760862	G/A	0.87	6.55E-05	0.847	0.000	G/A	0.604	0.86	1.20E-02	Imputed	1.00	G/A	0.601	0.87	1.71E-03	Imputed	0.98
12	rs59374718	74617548	A/G	0.74	3.49E-05	0.609	0.000	A/G	0.982	0.83	4.54E-01	Imputed	0.85	A/G	0.918	0.73	3.87E-05	Imputed	0.97
12	rs1020461	78571186	C/T	0.87	5.19E-05	0.658	0.000	G/A	0.407	0.88	3.63E-02	Genotyped	-	C/T	0.385	0.86	4.77E-04	Genotyped	-
12	rs75718479	97876906	C/A	1.28	1.71E-05	0.732	0.000	C/A	0.910	1.32	7.75E-03	Imputed	0.95	C/A	0.877	1.26	6.50E-04	Imputed	0.98
12	rs4964452	106607174	C/G	0.87	8.52E-05	0.916	0.000	C/G	0.589	0.88	2.83E-02	Imputed	0.98	C/G	0.573	0.87	1.26E-03	Imputed	1.00
12	rs9739933	128564339	G/A	1.23	8.30E-05	0.733	0.000	G/A	0.764	1.21	6.27E-03	Imputed	0.98	G/A	0.810	1.25	4.20E-03	Imputed	0.51
13	rs9580843	24620011	T/A	0.79	6.73E-05	0.164	0.483	T/A	0.902	0.70	6.42E-04	Imputed	0.96	T/A	0.894	0.83	1.24E-02	Imputed	0.90
13	rs9519977	106835388	A/G	0.73	6.84E-05	0.393	0.000	G/A	0.049	1.25	8.15E-02	Genotyped	-	A/G	0.952	0.70	2.46E-04	Imputed	0.96
13	rs615250	110808389	C/A	0.74	9.51E-05	0.722	0.000	A/C	0.050	1.42	1.27E-02	Imputed	1.00	C/A	0.949	0.75	2.57E-03	Imputed	1.00
14	rs17123969	51946458	A/G	1.20	5.26E-05	0.199	0.393	G/A	0.182	0.90	1.66E-01	Genotyped	-	A/G	0.802	1.26	6.42E-05	Imputed	0.95
14	rs58262369	64693912	C/T	0.73	3.83E-07	0.929	0.000	C/T	0.889	0.72	8.04E-04	Imputed	1.00	C/T	0.928	0.73	1.40E-04	Imputed	0.97
14	rs142159831	98420997	A/C	0.47	4.18E-05	0.409	0.000	A/C	0.984	0.55	2.09E-02	Imputed	0.94	A/C	0.994	0.40	4.73E-04	Imputed	0.95
15	rs6493618	53537453	C/T	1.20	1.53E-05	0.204	0.380	C/T	0.759	1.29	2.64E-04	Imputed	0.96	C/T	0.792	1.15	8.63E-03	Imputed	0.97
15	rs8035836	100742786	C/T	1.17	1.64E-05	0.184	0.433	C/T	0.284	1.25	4.33E-04	Genotyped	-	C/T	0.509	1.13	4.62E-03	Genotyped	-
17	rs4597375	4494815	T/C	1.15	9.03E-05	0.159	0.497	C/T	0.356	0.81	6.00E-04	Genotyped	-	T/C	0.624	1.11	1.74E-02	Imputed	1.00
17	rs4583311	53453103	C/T	1.16	3.88E-05	0.501	0.000	C/T	0.385	1.13	4.91E-02	Genotyped	-	C/T	0.304	1.19	2.31E-04	Genotyped	-
17	rs8082456	80062658	A/G	0.72	8.10E-05	0.652	0.000	A/G	0.956	0.76	6.23E-02	Imputed	0.95	A/G	0.949	0.70	4.85E-04	Imputed	0.83
18	rs144488018	20519236	A/C	1.50	2.15E-05	0.574	0.000	A/C	0.954	1.59	8.87E-04	Imputed	0.99	A/C	0.966	1.43	6.91E-03	Imputed	0.93
18	rs3095761	28147942	T/C	0.83	5.06E-05	0.888	0.000	T/C	0.877	0.82	3.20E-02	Imputed	1.01	T/C	0.798	0.84	6.38E-04	Imputed	1.00
18	rs4940840	56801257	C/T	0.82	1.43E-05	0.079	0.676	T/C	0.190	1.36	9.18E-05	Imputed	0.96	C/T	0.805	0.87	9.99E-03	Imputed	0.99
20	rs4815659	4095093	G/C	1.24	3.48E-05	0.080	0.673	C/G	0.145	0.71	8.29E-05	Imputed	0.94	G/C	0.854	1.16	2.88E-02	Imputed	0.88
22	rs36594	30334382	T/C	0.74	4.37E-06	0.253	0.234	C/T	0.094	1.49	2.68E-04	Imputed	0.92	T/C	0.922	0.79	2.47E-03	Imputed	0.94

[a]Based on the NCBI database, build 37.

[b]Effect/non-effect allele.

[c]The *P* values were combined by meta-analysis under a fixed-effect model.

[d]*P* value of Cochran's Q-test for the heterogeneity.

[e]Effect allele frequency in the controls.

[f]Imputation quality.

Supplementary Table 5. Summary of the association results of meta-analysis and replication 1 study

Chr	SNP	Poistion[a]	Meta-analysis			Replication 1		
			Allele[b]	OR	*P*[c]	EAF (case/control)[d]	OR	*P*[e]
1	rs76014269	180923595	G/A	1.28	3.84E-05	0.846/0.871	0.85	1.14E-02
1	rs11120266	214359179	T/C	1.16	3.08E-05	0.556/0.572	0.94	1.97E-01
2	rs6753841	34272597	A/C	0.84	3.14E-05	0.856/0.851	1.04	5.87E-01
2	rs13031082	51280251	A/G	1.21	6.36E-05	0.833/0.851	0.88	5.63E-02
2	rs354211	54932516	C/T	1.17	3.41E-05	0.800/0.817	0.90	9.87E-02
2	rs34824338	169934114	C/T	1.22	3.94E-05	0.915/0.900	1.16	6.35E-02
2	rs59817105	196486923	T/C	0.87	6.42E-05	NA	NA	NA
2	rs11886390	216924139	T/A	1.18	2.90E-05	0.781/0.771	1.06	3.47E-01
3	rs138301420	151135814	A/G	0.60	4.95E-05	0.981/0.976	1.25	1.96E-01
4	rs74832629	38385467	G/A	1.18	4.84E-06	0.428/0.411	1.04	3.10E-01
4	rs4446264	145137401	A/C	1.18	3.53E-05	0.735/0.728	1.04	5.27E-01
5	rs13154478	161749087	A/G	1.37	3.01E-05	0.913/0.916	0.96	6.69E-01
6	rs12192568	27740438	C/A	0.65	3.09E-05	0.984/0.978	1.40	6.54E-02
6	rs9351810	72103444	C/T	0.87	8.62E-05	0.589/0.611	0.91	7.98E-02
6	rs73773204	123952569	T/C	1.21	8.85E-05	0.824/0.819	1.03	6.90E-01
7	rs10274959	33218314	T/C	1.36	6.70E-05	0.907/0.909	0.98	8.46E-01
7	rs55932730	147551667	G/C	0.87	7.73E-05	0.571/0.580	0.96	4.48E-01
8	rs2167065	76560454	G/A	1.16	2.73E-05	0.669/0.653	1.07	1.88E-01
8	rs10282777	78068366	G/A	0.84	1.12E-05	0.765/0.759	1.03	5.91E-01
9	rs13300882	101561046	G/A	0.83	3.60E-05	0.998/0.998	1.09	8.76E-01
10	rs11251231	2454195	C/T	0.87	8.12E-05	0.617/0.616	1.00	9.33E-01
10	rs4749884	9644800	C/A	1.15	4.74E-05	0.473/0.437	1.15	4.87E-03
10	rs2152433	25152336	C/T	0.85	4.03E-05	0.726/0.737	0.95	3.55E-01
10	rs77598054	80244583	G/A	1.23	2.08E-05	NA	NA	NA
11	rs12791447	7556577	C/T	1.26	1.05E-05	0.122/0.098	1.27	2.73E-03
11	rs2015997	70664218	C/A	1.25	6.46E-05	0.122/0.117	1.05	5.35E-01
11	rs11603610	80668994	T/G	0.64	1.59E-05	0.959/0.958	1.04	7.36E-01
11	rs678618	95760862	G/A	0.87	6.55E-05	NA	NA	NA
12	rs59374718	74617548	A/G	0.74	3.49E-05	0.982/0.976	1.36	1.73E-01
12	rs1020461	78571186	C/T	0.87	5.19E-05	0.387/0.398	0.95	3.53E-01
12	rs75718479	97876906	C/A	1.28	1.71E-05	0.922/0.906	1.22	2.49E-02
12	rs4964452	106607174	C/G	0.87	8.52E-05	0.584/0.580	1.02	7.30E-01
12	rs9739933	128564339	G/A	1.23	8.30E-05	0.762/0.743	1.10	9.21E-02
13	rs9580843	24620011	T/A	0.79	6.73E-05	0.903/0.909	0.93	3.76E-01
13	rs9519977	106835388	A/G	0.73	6.84E-05	0.946/0.946	1.00	9.79E-01
13	rs615250	110808389	C/A	0.74	9.51E-05	0.946/0.943	1.05	6.50E-01
14	rs17123969	51946458	A/G	1.20	5.26E-05	0.835/0.821	1.10	1.34E-01

14	rs58262369	64693912	C/T	0.73	3.83E-07	0.872/0.898	0.77	1.05E-03
14	rs142159831	98420997	A/C	0.47	4.18E-05	0.983/0.978	1.28	1.87E-01
15	rs6493618	53537453	C/T	1.20	1.53E-05	0.287/0.43	0.50	4.67E-15
15	rs8035836	100742786	C/T	1.17	1.64E-05	0.315/0.296	1.09	1.16E-01
17	rs4597375	4494815	T/C	1.15	9.03E-05	0.683/0.664	1.09	1.10E-01
17	rs4583311	53453103	C/T	1.16	3.88E-05	0.397/0.413	0.93	1.86E-01
17	rs8082456	80062658	A/G	0.72	8.10E-05	0.500/0.499	1.00	9.99E-01
18	rs144488018	20519236	A/C	1.50	2.15E-05	NA	NA	NA
18	rs3095761	28147942	T/C	0.83	5.06E-05	0.866/0.878	0.90	1.76E-01
18	rs4940840	56801257	C/T	0.82	1.43E-05	0.808/0.804	1.03	6.38E-01
20	rs4815659	4095093	G/C	1.24	3.48E-05	0.853/0.857	0.98	7.33E-01
22	rs36594	30334382	T/C	0.74	4.37E-06	0.910/0.917	0.92	3.58E-01

[a]Based on the NCBI database, build 37.

[b]Effect/non-effect allele.

[c]The *P* values were combined by meta-analysis under a fixed-effect model.

[d]Effect allele frequency.

[e]Additive model in the logistic regression analysis.

NA, SNPs with a call rate < 90%.

SNP	Position[a]	Allele[b]	EAF[c]		OR[e]	*P*[e]
			Aggressive[d]	Non-aggressive		
rs4749884	9644800	C/A	0.493	0.466	1.11	0.210
rs12791447	7556577	C/T	0.119	0.126	0.93	0.590
rs75718479	97876906	C/A	0.931	0.911	1.32	0.087
rs58262369	64693912	C/T	0.871	0.869	1.01	0.917

[a]Based on the NCBI database, build 37.

[b]Effect/non-effect allele.

[c]Effect allele frequency.

[d]Aggressive prostate cancer is defined as Gleason score ≥ 7, or T-stage ≥ T3, or N+, or M+.

[e]Additive model in the logistic regression analysis.

Supplementary Table 6. Associations of four SNPs with aggressiveness of prostate cancer

Supplementary Table 7. Association results between SNPs in LD (r^2 > 0.70) with four SNPs and prostate cancer risk in the population of European descent

Chr	SNP	Position[a]	LD[b]	Allele[c]	EAF (case/control)[d]	OR[e]	*P*[e]
10	rs11256268	9637656	0.85	T/A	0.474/0.497	0.91	0.228
10	rs12572160	9637722	0.77	G/T	0.423/0.412	1.05	0.557
10	rs10795729	9638008	0.77	T/C	0.423/0.412	1.05	0.532
10	rs7913440	9640544	0.99	T/C	0.418/0.425	0.97	0.674
10	rs10905587	9641023	0.90	G/T	0.489/0.499	0.96	0.555
10	**rs4749884**	9644800	Index SNP	A/C	0.411/0.421	0.96	0.535
11	rs61890184	7547587	1.00	A/G	0.113/0.116	0.96	0.699
11	rs4287322	7550773	0.61	C/T	0.191/0.204	0.92	0.323
11	rs35714133	7553787	0.98	T/C	0.058/0.063	0.92	0.535
11	rs35811539	7554439	0.98	C/T	0.058/0.062	0.92	0.535
11	**rs12791447**	7556577	Index SNP	G/A	0.065/0.073	0.89	0.313
11	rs35250525	7560172	0.98	G/C	0.058/0.062	0.92	0.540
11	rs35182749	7560198	0.98	C/G	0.058/0.062	0.92	0.540
11	rs34193334	7562191	0.93	A/G	0.044/0.05	0.88	0.359
12	rs60807320	97873960	1.00	T/C	0.086/0.081	1.07	0.549
12	rs75019997	97875686	1.00	A/G	0.086/0.082	1.06	0.621
12	rs75187198	97876251	1.00	T/G	0.086/0.082	1.06	0.621
12	**rs75718479**	97876906	Index SNP	A/C	0.086/0.081	1.06	0.587
12	rs76647978	97877063	0.98	C/T	0.087/0.082	1.07	0.530
12	rs75315283	97877572	1.00	G/A	0.087/0.082	1.07	0.530
12	rs4762388	97879002	0.98	C/T	0.087/0.082	1.07	0.561
12	rs74642463	97879654	0.98	A/T	0.014/0.013	1.08	0.778
12	rs17027047	97882151	1.00	A/G	0.087/0.083	1.05	0.623
12	rs74866068	97882497	1.00	T/C	0.087/0.084	1.05	0.662
12	rs77731925	97882818	1.00	A/G	0.088/0.083	1.06	0.593
12	rs17027055	97885053	0.98	C/T	0.089/0.084	1.07	0.547
12	rs4762390	97886741	1.00	T/C	0.088/0.083	1.07	0.514
12	rs1609685	97887475	0.91	G/C	0.087/0.082	1.08	0.500
12	rs77303250	97887517	0.91	C/A	0.087/0.081	1.08	0.465
12	rs75458243	97888502	0.91	T/A	0.024/0.019	1.28	0.246
12	rs76252891	97888929	0.91	C/T	0.085/0.08	1.06	0.580
12	rs57298504	97889116	0.91	T/C	0.085/0.08	1.06	0.573
12	rs117112895	97905099	0.77	C/G	0.068/0.072	0.94	0.615
12	rs149496358	97905807	0.75	A/G	0.063/0.069	0.90	0.394
14	rs79133931	64687926	0.95	T/C	NA	0.24	0.199
14	rs78824180	64690610	0.98	A/G	NA	0.24	0.199
14	rs13136	64692906	0.76	A/T	0.069/0.077	0.89	0.305
14	**rs58262369**	64693912	Index SNP	T/C	NA		

14	rs115952301	64696785	0.98	T/C	NA
14	rs12437103	64707234	0.94	C/A	NA
14	rs114064455	64710354	0.94	A/T	NA
14	rs11850375	64711377	0.94	T/C	NA
14	rs79512176	64711658	0.94	C/G	NA
14	rs75027286	64717237	0.82	G/A	NA
14	rs3742614	64735496	0.74	A/G	NA
14	rs145855293	64738189	0.74	A/G	NA
14	rs28499370	64738255	0.72	C/G	NA
14	rs28485225	64738264	0.74	A/G	NA
14	rs12435278	64740349	0.75	C/G	NA
14	rs12435307	64740636	0.75	G/C	NA
14	rs12436020	64741404	0.75	G/A	NA
14	rs59216189	64743130	0.75	C/T	NA
14	rs59677654	64748604	0.75	A/G	NA
14	rs192744247	64755309	0.75	A/G	NA
14	rs3783735	64756020	0.75	T/C	NA
14	rs3783734	64756089	0.75	C/G	NA
14	rs78481919	64757580	0.75	C/A	NA
14	rs17226046	64759984	0.75	T/C	NA
14	rs3829768	64761594	0.75	G/A	NA
14	rs10483774	64763599	0.75	A/G	NA
14	rs17226060	64763874	0.75	G/A	NA
14	rs12436302	64766486	0.75	T/C	NA
14	rs12436325	64766592	0.75	G/C	NA
14	rs114635295	64771374	0.75	A/G	NA
14	rs77852880	64774602	0.71	C/T	NA
14	rs117353148	64774799	0.71	T/C	NA
14	rs12436533	64775094	0.71	C/T	NA
14	rs117839819	64777473	0.71	A/C	NA
14	rs141435464	64778309	0.71	C/T	NA
14	rs116525217	64784933	0.71	C/T	NA
14	rs140901695	64784988	0.71	C/T	NA
14	rs114249126	64788322	0.71	T/C	NA
14	rs75344118	64790176	0.71	A/G	NA
14	rs9788542	64792456	0.71	A/G	NA
14	rs9788467	64793078	0.71	C/T	NA
14	rs146085080	64794270	0.71	T/C	NA
14	rs9788441	64796426	0.71	G/A	NA
14	rs17226081	64798618	0.71	C/T	NA
14	rs1571512	64799037	0.71	G/C	NA
14	rs150080570	64799797	0.71	A/G	NA

14	rs79375985	64802278	0.71	T/C	NA
14	rs116982359	64803750	0.71	A/G	NA
14	rs36108515	64804432	0.71	T/G	NA
14	rs34422347	64804462	0.71	C/T	NA
14	rs12435338	64805752	0.71	G/C	NA
14	rs12435395	64805874	0.71	T/C	NA
14	rs117261417	64806027	0.71	G/A	NA
14	rs12434085	64807280	0.71	C/T	NA
14	rs77371998	64808349	0.71	G/A	NA
14	rs118185167	64808411	0.71	G/A	NA
14	rs118062637	64809701	0.71	C/T	NA
14	rs117695041	64809973	0.71	C/T	NA
14	rs139485472	64813707	0.71	T/G	NA
14	rs117747004	64818218	0.71	T/C	NA
14	rs147919054	64820201	0.71	A/G	NA
14	rs118063986	64826532	0.71	G/A	NA
14	rs34051517	64830517	0.71	C/G	NA
14	rs12437063	64830713	0.71	A/G	NA

[a]Based on the NCBI database, build 37.

[b]Linkage disequilibrium (r^2) between each SNP and the index SNP.

[c]Effect/non-effect allele.

[d]Effect allele frequency.

[e]Additive model in the logistic regression analysis.

NA, SNPs with minor allele frequency < 1%.

Supplementary Table 8. The mRNA expression levels of genes in prostate tumor tissue and paired normal tissue samples from TCGA dataset

Locus	Gene	RefGene ID	Expression levels of mRNA (RPKM, median)[a]		$P_{\text{t-test}}$
			Tumor tissues	Normal tissues	
11p15.4	*CYB5R2*	51700	6.99	7.94	1.12E-12
	EIF3F	8665	11.56	11.31	7.74E-05
	NLRP10	338322	-1.00	-0.79	3.15E-01
	NLRP14	338323	0.23	1.23	3.23E-09
	OLFML1	283298	7.08	8.09	8.45E-10
	OR10A3	26496	-0.80	-0.39	1.40E-02
	OR10A6	390093	-0.98	-0.76	2.66E-01
	OR5E1P	26343	-1.24	-1.08	4.31E-01
	OR5P2	120065	-1.11	-0.74	8.53E-02
	OR5P3	120066	-0.90	-0.71	4.20E-01
	OVCH2	341277	0.30	1.99	9.93E-14
	PPFIBP2	8495	9.57	10.23	1.89E-10
	RBMXL2	27288	-0.48	-0.35	3.89E-01
	SYT9	143425	4.42	6.22	1.63E-12
14q23.2	*AKAP5*	9495	5.54	5.39	2.68E-01
	ESR2	2100	3.50	3.15	2.77E-02
	HSPA2	3306	7.37	8.13	9.63E-08
	MTHFD1	4522	10.09	9.85	5.04E-05
	SGPP1	81537	9.19	9.02	9.82E-02
	SYNE2	23224	10.78	11.02	2.14E-02
	ZBTB1	22890	9.85	9.72	3.93E-02
	ZBTB25	7597	6.29	6.31	8.20E-01

[a]Expression levels of mRNA were log2 transformed.

Supplementary Table 9. Summary of functional annotation of four SNPs using data from HaploReg and RegulomeDB

Chr	SNP[a]	Position[b]	LD[c]	Histon marks		DNAse[f]	Proteins bound[g]	Motifs changed[h]	Gene	Annotation Score[i]	
				Promoter[d]	Enhancer[e]						
10	rs11256268	9637656	0.85					BATF,Irf	-	-	7
10	rs7913440	9640544	0.99					ELF1,NF-AT,STAT	-	-	6
10	rs10905587	9641023	0.90					8 altered motifs	-	-	6
10	**rs4749884**	9644800	1.00				MAFK	HNF4,Nanog,RXRA	-	-	3b
11	rs61890184	7547587	1.00		GM12878, HepG2			Hoxa5	*PPFIBP2*	Intronic	5
11	rs35714133	7553787	0.98		GM12878	Osteobl,NHDF-Ad		4 altered motifs	*PPFIBP2*	Intronic	5
11	rs35811539	7554439	0.98		GM12878			PU.1	*PPFIBP2*	Intronic	6
11	**rs12791447**	7556577	1.00		GM12878			Hic1	*PPFIBP2*	Intronic	6
11	rs35250525	7560172	0.98			4 cell types	FOXA1,GATA3,P300	Nr2e3	*PPFIBP2*	Intronic	4
11	rs35182749	7560198	0.98			HRE	FOXA1,GATA3,P300	RXRA,Rhox11,STAT	*PPFIBP2*	Intronic	3a
11	rs34193334	7562191	0.93			4 cell types	GATA2,GATA3,P300	5 altered motifs	*PPFIBP2*	Intronic	3a
12	rs199667213	97873871	0.95					9 altered motifs	*RMST*	Intronic	6
12	rs60807320	97873960	1.00					AP-2,Evi-1,Znf143	*RMST*	Intronic	6
12	rs75019997	97875686	1.00			HA-sp			*RMST*	Intronic	5
12	rs75187198	97876251	1.00					12 altered motifs	*RMST*	Intronic	6
12	**rs75718479**	97876906	1.00					7 altered motifs	*RMST*	Intronic	7
12	rs76647978	97877063	0.98						*RMST*	Intronic	7
12	rs75315283	97877572	1.00						*RMST*	Intronic	7
12	rs4762388	97879002	0.98					TEF-1	*RMST*	Intronic	6
12	rs74642463	97879654	0.98		H1			Mef2,Rad21	*RMST*	Intronic	7
12	rs17027047	97882151	1.00					Foxo,YY1	*RMST*	Intronic	7
12	rs74866068	97882497	1.00					AIRE,Mef2,YY1	*RMST*	Intronic	6
12	rs77731925	97882818	1.00					CEBPB,p300	*RMST*	Intronic	5
12	rs17027055	97885053	0.98			Ishikawa			*RMST*	Intronic	5
12	rs4762390	97886741	1.00					Hand1	*RMST*	Intronic	7
12	rs1609685	97887475	0.91						*RMST*	Intronic	6
12	rs77303250	97887517	0.91					SIX5	*RMST*	Intronic	6

12	rs75458243	97888502	0.91					11 altered motifs	*RMST*	Intronic	6
12	rs76252891	97888929	0.91					10 altered motifs	*RMST*	Intronic	6
12	rs57298504	97889116	0.91					Nkx3	*RMST*	Intronic	5
14	rs79133931	64687926	0.95		K562				*SYNE2*	Intronic	7
14	rs78824180	64690610	0.98			WI-38		AP-1,Cphx	*SYNE2*	Intronic	5
14	**rs58262369**	64693912	1.00			HSMMtube,Osteobl,HRE		EWSR1-FLI1,STAT	*ESR2*	3'-UTR	5
14	rs115952301	64696785	0.98					Egr-1,Irf,SP1	*ESR2*	Intronic	5
14	rs12437103	64707234	0.94					4 altered motifs	*ESR2*	Intronic	7
14	rs114064455	64710354	0.94						*ESR2*	Intronic	7
14	rs11850375	64711377	0.94		NHLF	BE2_C		Spz1	*ESR2*	Intronic	5
14	rs79512176	64711658	0.94					NRSF,PU.1,Pbx3	*ESR2*	Intronic	6
14	rs75027286	64717237	0.82			HA-h		RREB-1	*ESR2*	Intronic	5

[a]Index SNPs were depicted in bold.

[b]Based on the NCBI database, build 37.

[c]LD was defined as $r^2 > 0.80$.

[d]Histone modifications of H3K4me1 and H3K27ac.

[e]Histone modification of H3K4me3.

[f]The levels of DNase I hypersensitivity.

[g]The binding of transcription factor.

[h]The alteration in regulatory motif.

[i]RegulomeDB score.

Supplementary Table 10. Analysis of eQTL in newly identified loci associated with prostate cancer

Locus	SNP	Position	TCGA eQTL			Blood eQTL brower	
			Gene	RefGene ID	P_{additive}	Gene	P_{additive}
11p15.4	rs12791447	7556577	*CYB5R2*	51700	0.438		
			EIF3F	8665	0.051		
			NLRP10	338322	0.339		
			NLRP14	338323	0.035		
			OLFML1	283298	0.609		
			OR10A3	26496	0.978		
			OR10A6	390093	0.368		
			OR5E1P	26343	0.878		
			OR5P2	120065	0.194		
			OR5P3	120066	0.805		
			OVCH2	341277	0.496		
			PPFIBP2	8495	0.024	*PPFIBP2*	1.16E-21
			RBMXL2	27288	0.739		
			SYT9	143425	0.858		
14q23.2	rs58262369	64693912	*AKAP5*	9495	0.549		
			ESR2	2100	0.482		
			HSPA2	3306	0.093		
			MTHFD1	4522	0.346		
			SGPP1	81537	0.586		
			SYNE2	23224	0.902		
			ZBTB1	22890	0.574		
			ZBTB25	7597	0.545		

Supplementary Table 11. Allele frequency of four SNPs among different ethnics from the 1000 Genomics Project

Locus	SNP	Position[a]	Allele	Frequency in four populations[b]			
				CHB	JPT	CEU	YRI
10p14	rs4749884	9644800	C	0.526	0.562	0.577	0.989
			A	0.474	0.438	0.424	0.011
11p15.4	rs12791447	7556577	C	0.088	0.157	0.071	0.000
			T	0.912	0.843	0.929	1.000
12q23.1	rs75718479	97876906	C	0.892	0.933	0.929	1.000
			A	0.108	0.067	0.071	0.000
14q23.2	rs58262369	64693912	C	0.861	0.933	1.000	0.926
			T	0.139	0.067	0.000	0.074

[a]Based on the NCBI database, build 37.

[b]CHB , Han Chinese in Beijing, China; JPT, Japanese in Tokyo, Japan; CEU, (CEPH) with Northern and Western European ancestry; YRI, Yoruba in Ibadan, Nigeria.

Supplementary Table 12. Sequences of primers and probes of 4 SNPs in the TaqMan assay

SNP	Sequence (5'-3')
rs4749884	F: CACCAAATATGGCTTCGTGATC
	R: GATGAACTATGCCCTTGGCTTCT
	PROBE-C: FAM-TGACCCCAGCAGCA-MGB
	PROBE-A: HEX-ACCCCAGAAGCAGA-MGB
rs12791447	F: CCTTTAGTTGAGGAAACAGCATGA
	R: GCCGCAAGAGAGCATCCTT
	PROBE-C: FAM-GCCACCGTGCCTTT-MGB
	PROBE-T: HEX-TGCCACTGTGCCTTT-MGB
rs75718479	F: GAATGAATGAATGACTAGGCCACTG
	R: GCGTGGTTCTTTAGCCCTTTATTAG
	PROBE-A: FAM-TTTTTAGTTTATTGGCCCTTT-MGB
	PROBE-C: HEX-ATTTTTAGTTTCTTGGCCCTT-MGB
rs58262369	F: CTGGTTTCCCCTGAGGTTTG
	R: GGCACAGCTGACCACACAAT
	PROBE-C: FAM-CCTTCTCTGCCAAA-MGB
	PROBE-T: HEX-CCTTCTTTGCCAAA-MGB
